# Detection of SARS-CoV-2 in Milk From COVID-19 Positive Mothers and Follow-Up of Their Infants

**DOI:** 10.3389/fped.2020.597699

**Published:** 2020-10-27

**Authors:** Enrico Bertino, Guido Eugenio Moro, Giuseppe De Renzi, Giuseppina Viberti, Rossana Cavallo, Alessandra Coscia, Carlotta Rubino, Paola Tonetto, Stefano Sottemano, Maria Francesca Campagnoli, Antonella Soldi, Michael Mostert, Francesco Cresi, David Lembo, Collaborative Research Group on SARS-CoV-2 in Human Milk

**Affiliations:** ^1^Neonatal Care Unit of the University, City of Health and Science Hospital, Turin, Italy; ^2^Italian Association of Human Milk Banks, Milan, Italy; ^3^Laboratory of Clinical Pathology and Microbiology, San Luigi Gonzaga Hospital, Turin, Italy; ^4^Department of Public Health and Pediatric Sciences, University of Turin, Turin, Italy; ^5^Microbiology and Virology Unit, City of Health and Science, Turin, Italy; ^6^Neonatal Care Unit, City of Health and Science University Hospital of Turin, Turin, Italy; ^7^Laboratory of Molecular Virology, Department of Clinical and Biological Sciences, University of Turin, Turin, Italy

**Keywords:** breastfeeding, human milk, newborn, COVID-19, pandemic

## Abstract

**Background:** In the current SARS-Coronavirus-2 (SARS-CoV-2) pandemic little is known about SARS-CoV-2 in human milk. It is important to discover if breast milk is a vehicle of infection.

**Objective:** Our aim was to look for the presence of SARS-CoV-2 RNA in the milk of a group of SARS-CoV-2 positive mothers from North-West Italy.

**Methods:** This is a prospective collaborative observational study where samples of human milk from 14 breastfeeding mothers positive for SARS-CoV-2 were collected. A search of viral RNA in breast milk samples was performed by RT-PCR (Real-Time reverse-transcriptase-Polymerase-Chain-Reaction) methodology tested for human milk. All the newborns underwent a clinical follow up during the first month of life or until the finding of two sequential negative swabs.

**Results:** In 13 cases the search for SARS-CoV-2 RNA in milk samples resulted negative and in one case it was positive. Thirteen of the 14 newborns were exclusively breastfed and closely monitored in the first month of life. Clinical outcome was uneventful. Four newborns tested positive for SARS-CoV-2 and were all detected in the first 48 h of life, after the onset of maternal symptoms. Also the clinical course of these 4 infants, including the one who received mother's milk positive for SARS-CoV-2, was uneventful, and all of them became SARS-CoV-2 negative within 6 weeks of life.

**Conclusion:** Our study supports the view that SARS-CoV-2 positive mothers do not expose their newborns to an additional risk of infection by breastfeeding.

## Introduction

In the current SARS-Coronavirus-2 (SARS-CoV-2) pandemic it is important to identify every possible route of transmission to prevent the spread of the disease. To date, all reports suggest that vertical transmission of SARS-CoV-2 is unlikely (1–6). However, the possible transmission of the virus via breastfeeding is still under debate. Providing reliable data on this issue may have significant practical implications for the management of the SARS-CoV-2 positive mother-infant dyad and breastfeeding support. Based on current knowledge from clinical studies, it appears unlikely that the virus is transmitted through breast milk (1, 2, 4, 7–11). However, the data on the detection of SARS-CoV-2 in breast milk available in the literature, have a low sample size (12–14) and in most cases methodological limitations (15).

Here, we report the results of a prospective collaborative observational study whose primary aim was to look for the presence of SARS-CoV-2 RNA in the milk of a group of SARS-CoV-2 positive mothers from North-West Italy, an area with a very high incidence for Coronavirus disease 19 (COVID-19) at the time of sample collection. The study was specifically designed to analyze human milk, with the adoption of a real-time PCR method tested to effectively detect SARS-COV-2 nucleic acid in human milk, a fluid that is difficult to analyze due to its complex composition. The secondary aim of the study was to evaluate clinical outcome during the first month of life in infants exclusively breastfed by their positive mothers.

## Methods

### Study Design

This is a prospective collaborative observational study coordinated by the Neonatal Care Unit and the Laboratory of Molecular Virology of the University of Turin, Italy.

The study was approved by the local Ethical Committee (protocol number 0039684), and informed consent to participate in the study was obtained from each mother.

The study centers were located in North–West Italy. From April 1st 2020 to July 31st 2020, these centers collected samples of milk from 14 breastfeeding mothers diagnosed with Covid-19.

All the mothers were diagnosed with COVID-19 by real-time reverse-transcriptase–polymerase-chain-reaction (RT-PCR) assays of nasal and pharyngeal swabs, in accordance with WHO guidance (16). We collected clinical records, breastfeeding history, and laboratory findings related to SARS-CoV-2 of 14 mothers and their infants during hospital stay and in the 6 weeks after discharge.

Newborn follow up was performed during the first month of life or until the finding of two sequential negative swabs.

### Sample Collection

Milk collection was performed with a dedicated breast-pump in 10 of the mothers and in four with manual expression. All mothers were advised to follow strict hygienic rules according to international recommendations (17, 18). For milk expression mothers wore a disposable surgical face mask, washed their hands with water and soap for at least 20 s, cleaned their breast with warm water and soap and then dried it with a clean paper towel. Immediately after breast milk was collected and transferred in sterile single-use plastic containers and stored in a freezer at −20°C. Eight mothers expressed their milk in the hospital (cases 4, 6, 8, 9, 11, 12, 13, 14), five mothers expressed it at home (cases 1, 2, 5, 7, 10), and one mother (case 3) expressed the first two samples in the hospital and the following four samples at home.

### Detection Methods of SARS-CoV-2 in Human Milk by RT-PCR

Analysis of the milk samples was carried out by the Laboratory of Clinical Microbiology at the San Luigi Gonzaga Hospital, Orbassano, Italy. Positivity to SARS-CoV-2 in milk samples from Case 3 was confirmed by the Microbiology and Virology Unit at City of Health and Science of Turin, Italy. Before analyzing milk samples, the analytical methods were tested for use in milk. To this aim, the SARS-CoV-2 positive control plasmid provided by the manufacturer of the analytical kits, was added to a milk sample collected in February 2019 from an Italian woman, almost 1 year before the onset of the COVID-19 outbreak in Italy and therefore surely negative for SARS-CoV-2. In both laboratories the positive control was properly detected by RT-PCR generating a standard amplification curve and a threshold cycle similar to that obtained when the analytical methods were used in routine diagnostic procedures. At the San Luigi Gonzaga Hospital RNA was extracted from milk specimens using the BD MAX™ ExK™ TNA-3 and determined by rRT-PCR targeting the SARS-CoV-2 S gene by using the VIASURE SARS-CoV-2 Real Time PCR Detection Kit (CertTest, Zaragoza, Spain). Real-Time qPCR reactions were performed on BD MAX RT-PCR apparatus (Becton Dickinson). At the Microbiology and Virology Unit of City of Health and Science of Turin, extraction was carried out with the QIAsymphony DSP Virus/Pathogen Midi Kit (Qiagen) and amplification with the Seegene AllplexTM 2019-nCoV Assay (target genes: E, N, RdRp).

## Results

The main clinical characteristics of our population and the results of SARS-CoV-2 search in milk samples are summarized in Table 1.

**Table 1:** Characteristics of the study population and results of SARS-CoV-2 search in milk samples.

	**Maternal age (years)**	**Gestational age at delivery (weeks)**	**Method of delivery**	**Interval between delivery and milk collection (days)**	**Interval between maternal positive swab and milk collection (days)^**°**^**	**Maternal symptoms at milk collection**	**Breast milk test**	**First maternal positive swab (days before (–) or after (+) delivery)**	**First maternal negative swab (days after delivery)**
Case 1	25	40	Vaginal	27	0	No	Negative	+3	41
Case 2^□^	28	37	Vaginal	14	0	No	Negative	0	25
Case 3^□^	26	38	CS	5	4	Yes	Positive	+1	34
Case 4^*^	30	36	Vaginal	4	5	Yes	Negative	−1	23
Case 5	34	38	CS	21	6	No	Negative	0	24
Case 6	35	30	CS	3	0	Yes	Negative	−8	33
Case 7	30	39	CS	27	0	No	Negative	+1	45
Case 8^**^	38	32	CS	2	12	Yes	Negative	−9	3
Case 9^□^	24	34	CS	4	5	No	Negative	0	9
Case 10	29	41	Vaginal	13	10	No	Negative	−1	16
Case 11	37	38	CS	4	5	No	Negative	−11	16
Case 12	31	40	Vaginal	3	3	Yes	Negative	0	23
Case 13	37	37	CS	1	0	Yes	Negative	−18	20
Case 14	32	37	CS	3	0	No	Negative	−1	14

* *Mother decided to stop breastfeeding at discharge (day 5)*.

** *Mother with interstitial pneumonia. Ritonavir/Lopinavir therapy discontinued 3 days before the milk collection*.

□ *Manual expression*.

° *We considered the time interval as 0 if the milk collection took place between the recording of two positive maternal swabs*.

Eleven out of 14 mothers were symptomatic at the moment of the test for SARS-CoV-2, performed in eight cases just before delivery and in three cases after delivery and presented at least one of following signs: fever, myalgia, rhinitis, cough, dyspnea, sore throat, conjunctivitis, diarrhea, chest pain, anosmia, ageusia. In three asymptomatic mothers (cases 9, 11, 14), the nasopharyngeal swab for SARS-CoV-2 was performed as a routine test at hospital admission (required in two centers).

In thirteen cases the assays were negative for SARS-CoV-2 RNA and in one case it was positive (case 3).

### Neonatal Outcome

In our population, four newborns tested positive for SARS-CoV-2 (cases 1, 3, 7, 9 in Table 1), and were all detected in the first 48 h of life, after the onset of maternal symptoms and the positivity of a swab test. The clinical course of these four infants was carefully monitored and was uneventful and all of them became negative by 6 weeks of life. All of them received exclusive breastfeeding during the observational period.

The other 10 infants tested negative for SARS-CoV-2. Nine of them were breastfed, and one received artificial formula from fourth day of life (the breast milk sample was collected previously). None of them turned out to be positive and/or symptomatic during the first month of life.

### Case 3 Report

**Figure 1:**
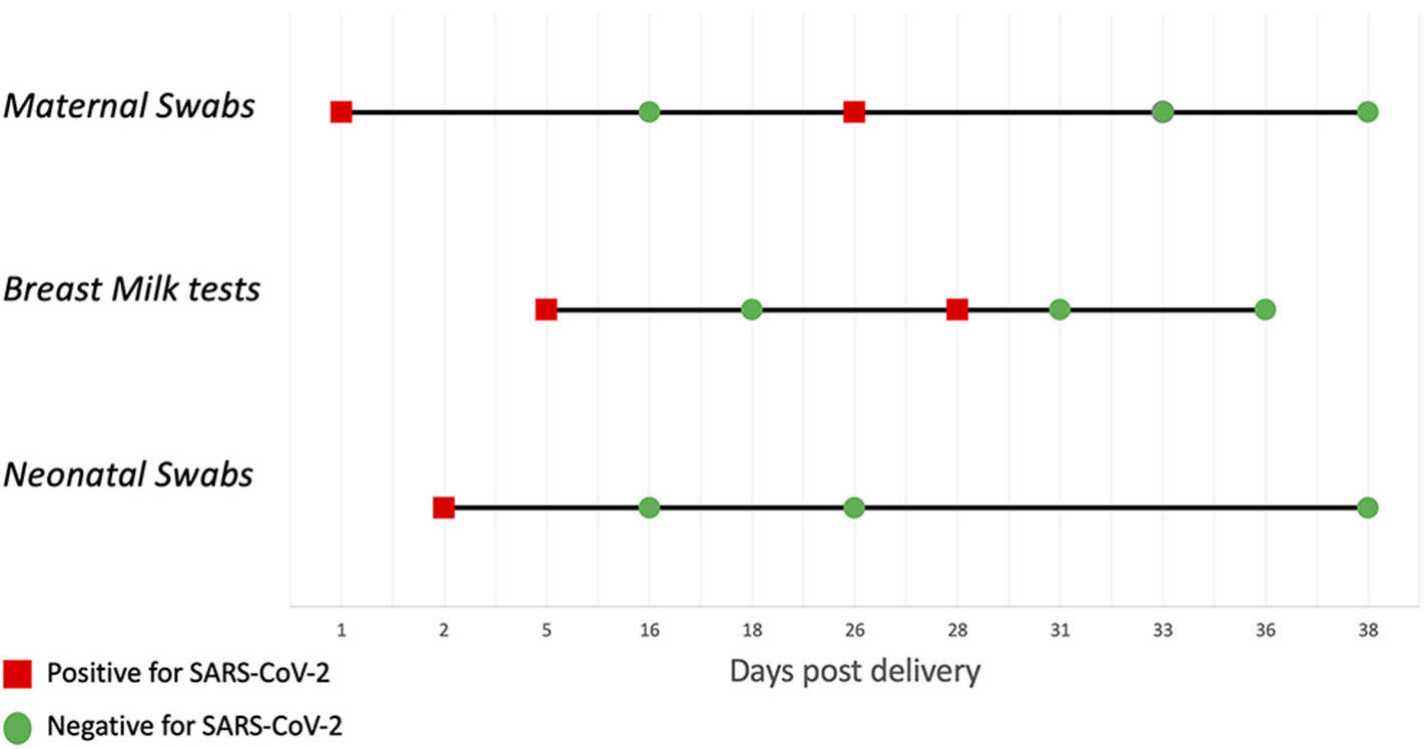
Case 3: swabs and breast milk tests trend.

Case 3 resulted positive for SARS-CoV-2 at the first two milk collections, based on two different samples collected with manual expression with 1-h interval at day 5 after delivery. Further samples were collected at day 18, 28, 31 and 36 after delivery. Of these samples, only the one collected at 28 days resulted weakly positive for SARS-CoV-2 (Figure 1). The mother and her infant were kept together immediately after delivery and practiced rooming-in in the first 2 days of life without any specific precautions, until the first clinical signs of COVID-19 appeared in the mother (fever, cough, ageusia, anosmia) 1 day after delivery. A maternal nasopharyngeal swab was performed and resulted positive for SARS-CoV-2. The day after a nasopharyngeal swab was performed in the newborn, with a positive result for SARS-CoV-2 as well. Maternal fever and coughing disappeared in 1 week, with residual anosmia and ageusia still persisting after 1 month. The newborn was exclusively breastfed for the first month of life, closely monitored during this period and the clinical outcome was uneventful. The newborn was found negative for SARS-CoV-2 at two consecutive nasopharyngeal swabs at day 16 and 26. One more swab was performed and resulted negative at 38 days of life (because the mother was again positive at day 26).

## Discussion

In this case series we found that milk samples of 13 out of 14 SARS-CoV-2 positive mothers were negative for the virus. In one case the milk samples tested positive. Thirteen out of the 14 newborns were exclusively breastfed during the observation period, and clinical outcome in all of them was carefully monitored and was uneventful. Thus, we have no evidence to consider breastfeeding in SARS-CoV-2 positive mothers as unsafe for their newborns. A recent cohort study (11) concerning breastfed infants of SARS-CoV-2 positive mothers in the first month of life, revealed that they remained SARS-CoV-2 negative on nasopharyngeal swab. However, the presence of the virus in human milk was not evaluated. Recently the presence of SARS-CoV-2 in breastmilk was evaluated in 18 infected women from delivery to 19 months, with only one sample found positive (19). These data are in accordance with our results.

We suppose that in the four newborns who tested positive for SARS-CoV-2, the virus passed from the mother to the infant through intrapartum or airborne exposure. The four infants remained asymptomatic during the observation period including case 3, in whom, irrespective of the reason for milk positivity, careful monitoring did not reveal any clinical symptoms. Of particular interest is that this infant at 16 and 26 days tested negative although the mother was positive again at 26 days and the milk was again positive at 27 days (Figure 1). This supports the view that breast milk, even when positive, is not a route of contagion.

A recently published letter (12) describes a case where milk samples of a mother with COVID-19 were positive for SARS-CoV-2, and the newborn after discharge was re-hospitalized with icterus and respiratory symptoms. As this newborn tested positive for both Respiratory Syncytial Virus (RSV) and SARS-CoV-2, it is possible that the symptoms for re-hospitalization were due to RSV and not to SARS-CoV-2, being the former a common cause of bronchiolitis. However, even if the newborn symptoms were Covid-19 related, this does not mean that the oral route of transmission poses an additional risk respect to the respiratory route. In our study, we hypothesize three possible explanations for the positivity of SARS-CoV-2 in breast milk of case 3.

The first is that the virus could have shed in the milk of the infected mother during lactation. The argument supporting this hypothesis is that the virus was consistently detected in samples from three independent collections (Figure 1).

Furthermore, the pattern of positivity in milk samples was consistent with that of the mother's swabs. On the basis of the literature available so far, including this report, the presence of SARS-CoV-2 in human milk is a rare event. Indeed, up to now there is no evidence of shedding, apart from a case of SARS-CoV-2 in human milk that was detected, but not confirmed when retested 2 days after (3) and three cases in which it was consistently found positive (12–14). Data on other human coronaviruses do not shed light on this issue. A single case report failed to detect SARS-CoV in the milk of a SARS positive mother (20), while no data are available on MERS-CoV. A study on the human coronavirus NL63, causing common cold, provided limited evidence of vertical transmission and did not investigate the presence of viral RNA in milk samples (21).

The second hypothesis is that milk samples were contaminated in the laboratory during the assay procedures. This possibility is highly unlikely since the virus was found in three samples from the same mother analyzed independently.

The third hypothesis is that milk was contaminated due to an inadequate compliance with hygiene measures in expressing the milk.

Finally, detection of SARS-CoV-2 RNA does not necessarily imply the presence of an infectious and active virus that can be transmitted via breastfeeding to infect the infant (19).

In conclusion, our study supports the view that SARS-CoV-2 positive mothers do not expose their newborns to an additional risk of infection by breastfeeding. This is an important message that should be given to mothers and healthcare providers in this moment of uncertainty. This information is useful also for all mothers living in areas with high prevalence of COVID-19 infection. In fact, our results support the view that they should breastfeed, irrespective of swab test results, considering the immunological and anti-infective properties of mother's milk. Clearly, the recommended hygiene measures for the control of airborne exposure, for direct breastfeeding, as well as for milk expression when a mother and her infant need to be separated, must be carefully followed.

Findings from our study are limited by small samples size and self-collection of milk samples so future studies are needed to confirm our results.

## Data Availability Statement

The raw data supporting the conclusions of this article will be made available by the authors, without undue reservation.

## Ethics Statement

The studies involving human participants were reviewed and approved by Comitato Etico Interaziendale A.O.U. Città della Salute e della Scienza di Torino - A.O. Ordine Mauriziano - A.S.L. Città di Torino. Written informed consent to participate in this study was provided by the participants' legal guardian/next of kin. Written informed consent was obtained from the individual(s), and minor(s)' legal guardian/next of kin, for the publication of any potentially identifiable images or data included in this article.

## Author Contributions

DL, EB, and GEM conceived the study, analyzed the data, and wrote the manuscript. GDR, GV, and RC performed the RT-PCR assays. AC, CR, PT, and MM contributed to writing the manuscript. AC, FC, CR, PT, SS, AS, MC, and the other members of the Collaborative Research Group collected the samples and performed the clinical follow up. All authors read and approved the final manuscript.

## Conflict of Interest

The authors declare that the research was conducted in the absence of any commercial or financial relationships that could be construed as a potential conflict of interest.
